# Functional outcomes after resections for low rectal tumors: comparison of Transanal with laparoscopic Total Mesorectal excision

**DOI:** 10.1186/s12893-019-0550-4

**Published:** 2019-07-05

**Authors:** Mateusz Rubinkiewicz, Piotr Zarzycki, Jan Witowski, Magdalena Pisarska, Natalia Gajewska, Grzegorz Torbicz, Michał Nowakowski, Piotr Major, Andrzej Budzyński, Michał Pędziwiatr

**Affiliations:** 1 2nd Department of General Surgery, Jagiellonian University Medical College, Kopernika 21, 31-501 Kraków, Poland; 2 Centre for Research, Training and Innovation in Surgery (CERTAIN Surgery), Kraków, Poland

**Keywords:** Functional outcomes, Faecal incontinence, Transanal approach, Low anterior resection syndrome, Total mesorectal excision

## Abstract

**Background:**

Aim of this study was to evaluate functional outcomes of transanal total mesorectal excision (TaTME) in comparison to conventional laparoscopic approach (LaTME) in terms of low anterior resection syndrome (LARS).

**Methods:**

Forty-six patients who underwent total mesorectal excision for low rectal cancer between 2013 and 2017 were enrolled. Primary outcome was the severity of faecal incontinence, assessed both before the treatment and 6 months after ileostomy reversal. LARS score and Jorge-Wexner scale were utilized to analyze its severity.

**Results:**

Twenty (87%) from TaTME and 21 (91%) from LaTME group developed LARS postoperatively. There were no significant differences between groups in terms of LARS occurrence (*p* = 0.63) and severity. The median Wexner score was comparable in both groups (8 [IQR: 4–12] vs 7 [3–11], *p* = 0.83). Univariate analysis revealed that postoperative complications were a risk factor for LARS development (*p* = 0.02). Perioperative outcomes, including operative time, blood loss and intraoperative adverse events did not differ significantly between groups either. Five TaTME patients developed postoperative complications, while there were morbidity 6 cases in LaTME group. Quality of mesorectal excision was comparable with 20 and 19 complete cases in TaTME and LaTME groups, respectively.

**Conclusions:**

TaTME provided comparable outcomes in terms of functional outcomes in comparison to LaTME for total mesorectal excision in low rectal cancers. Having said that, LARS prevalence is still high and requires further evaluation of the technique.

## Background

Low anterior resection with primary anastomosis is frequently associated with a set of postoperative symptoms, referred to as low anterior resection syndrome (LARS) [1]. The incidence and severity of LARS varies in different studies. It can reach up to 90% in patients undergoing rectal cancer treatment, among whom up to 60% will develop major LARS [2–4]. Among suggested etiologic factors, damage of autonomic nerves related to neoadjuvant radiation therapy and surgical procedures are mentioned [5].

Introduction of minimally invasive techniques (either laparoscopic or robotic) to rectal cancer surgery improved short-term clinical outcomes, including reduced length of stay (LOS), surgical site infection rate and postoperative pain, while maintaining similar oncological outcomes [6–9]. Although this approach facilitates better visualization of the operative field, especially regarding pelvic nerves, no improvement in terms of LARS has been noted so far [10].

A novel, transanal approach to total mesorectal excision (TaTME) is believed to be at least non-inferior alternative in terms of oncological results for open and laparoscopic resections [11]. However, concerns have been risen about the influence of transanal approach, combined with extensive dissection of pelvic structures, on postoperative function of anal sphincters [12]. This aspect of TaTME has not been studied so far.

We designed this study to evaluate functional outcomes of TaTME in comparison to conventional laparoscopic total mesorectal excision (LaTME) in terms of LARS.

## Methods

### Setting and design

Between 2013 and 2017 a prospective database of patients undergoing total mesorectal excision for rectal cancer was developed. All subjects were patients in tertiary referral university hospital with annual volume of approximately 50 cases. TaTME was introduced in 2014 year. In 2015, TaTME technique was introduced to the department’s clinical practice as a standard procedure. We excluded first 10 cases from the analysis due to avoid bias related to the learning curve. We are COLOR III study approved centre. LaTME group was recruited from patients operated between 2013 and 2015. Only patients with low rectal cancer (up to 5 cm from the anal verge) were included. Patients requiring abdominoperineal resection (cancer infiltration on external anal sphincter persistent after neoadjuvant chemoradiotherapy) were excluded from the analysis. Chemoradiotherapy was used in patients initially assessed as T3 and T4 or N+ in MRI examination. We routinely use long courses of radiotherapy with a total dose of 50.4 Gy and concomitant chemotherapy, which contains 38-day course of capecitabine. The resection is scheduled 8 weeks after the neoadjuvant treatment completion. All procedures were performed by a team of experienced surgeons with expert skills in laparoscopic surgery, who had been trained in TaTME technique during cadaver-based courses. During the study period, the main operator was the same surgeon in all cases.

For the purpose of this study, faecal incontinence was assessed twice: before the start of the neoadjuvant treatment and 6 months after ileostomy reversal. To stratify its severity, we used LARS score and Jorge-Wexner scale [1, 13]. The questionnaires were collected prospectively by a member of surgical team during a visit in an outpatient clinic. Clavien-Dindo scale was used for reporting perioperative complications [14].

### Operative technique

The method of performing LaTME implemented in our department is described elsewhere [15]. In TaTME cases, we used a modified technique with application of Karl Storz TEO platform instead of GelPOINT Path Trananal Access Platform, that is used by most teams. We routinely use one-team approach. The anastomoses were performed with circumferential mechanical stapler or were hand sewn in case it was impossible to use stapler due to low level of the anastomosis. In TaTME patients, the purse string suture before anastomosis was done with TEO TEM platform. 33 or 31 mm circumferential stapler was used, depending on the bowel diameter. In all cases straight anastomosis was performed, with no modifications such as coloplasty or j-pouch. Defunctioning ileostomy was executed in all cases. In case of manifestation of anastomotic leakage (clinical, radiological or endoscopic), laparoscopic lavage with pelvic drainage was performed. Also, intraoperative colonoscopy was done with application of EndoVac negative pressure wound treatment therapy. If there was no clinical evidence of anastomotic leakage, a colonoscopic examination was scheduled 3 weeks after initial surgery, in order to confirm proper healing of the anastomosis. Additionally, the test of the anal sphincter function was performed (digital rectal examination, liquid continence after enema application). The date of ileostomy reversal was scheduled when there was no leakage found. Every patient was treated according to Enhanced Recovery After Surgery protocol [16, 17].

### Measured outcomes

Primary outcome was the severity of fecal incontinence measured with LARS score and Jorge-Wexner scale. The assessment was done twice – before the treatment and 6 months after ileostomy reversal. LARS was classified as major when patient scored at least 30 points and as minor with 21–29 points [1]. Secondary outcomes were perioperative features: operative time, blood loss, number of intraoperative adverse effects, number of complications and pathological quality of the resected specimen. Pathological assessment was performed according to Quirke criteria [18].

### Statistical analysis

All data were analyzed with Statistica version 13.0 PL (StatSoft Inc., Tulsa, OK, USA). Continuous results are presented as median and interquartile range (IQR). Categorical variables were compared by chi-square test. The Shapiro-Wilk test was used to check for normal distribution of data, and Student’s *t*-test was used for normally distributed quantitative data. For non-normally distributed quantitative variables, Mann-Whitney *U* test was used. A *p*-value < 0.05 was considered statistically significant. All considerable patient- and treatment-related factors were analyzed with logistic regression models in search of risk factors for LARS.

The study was approved by the local ethics committee. Each patient signed an informed consent before inclusion in the study.

## Results

Fourty-six patients were included to the analysis. Demographic characteristics are presented in Table 1.

**Table 1:** Demographical characteristics

	LaTME	TaTME	*p* value
Number of patients	23	23	–
Age (median, IQR)	64 [58–67]	60 [51–67]	0.41
Sex (women)	10 (31%)	10 (31%)	0.37
BMI (kg/m^2^; median, IQR)	26.5 [23.8–30.6]	26 [22.8–29.7]	0.43
Distance of the tumor from the anal verge (cm; median, IQR)	4 [3–5]	3[2–4]	0.01
ASA I/II/III	2/16/5	3/15/6	0.60
Preoperative neoadjuvant treatment (yes/no)	19/4	18/5	0.71
Adjuvant treatment (yes/no)	15/8	13/10	0.54
Type of anastomosis (stapled/hand-sewn)	21/2	21/2	1.00
Clinical TNM before neoadjuvant treatment			
cT1/2/3/4	3/6/12/2	2/3/15/3	0.24
cN	13/23	14/23	0.76
Postoperative LARS incidence			
No LARS	2 (9%)	3 (13%)	0.63
Minor LARS	9 (39%)	12 (52%)	0.37
Major LARS	12 (52%)	8 (35%)	0.23
Preoperative LARS Score (median, IQR)	0 [0–5]	5 [0–21]	0.10
Postoperative LARS score (median, IQR)	30 [21–34]	29 [24–34]	0.76
Preoperative Wexner (median, IQR)	0 [0–1]	0 [0–2]	0.20
Postoperative Wexner (median, IQR)	7 [3–11]	8 [4–12]	0.83

Median preoperative LARS score were 0 (IQR: 0–5) and 5 (0–21) in LaTME and TaTME groups, respectively. There were no significant differences between the groups (*p* = 0.10). Median Wexner score was 0 (0–1) in LaTME and 0 (0–2) in TaTME group and the results were also not statistically significant (*p* = 0.20). None of the patients in LaTME group suffered from fecal incontinence. Two TaTME patients had minor, and one patient minor LARS preoperatively.

In postoperative evaluation, 20 (87%) patients from TaTME group developed LARS, whereas in LaTME group 21 (91%) patients were affected by it (*p* = 0.63). 8 patients (35%) in TaTME group developed major LARS, whereas there were 12 individuals (52%) in LaTME group. There were no significant differences between groups (*p* = 0.23). 12 patients (52%) in TaTME group and 9 (39%) patients in LaTME group had minor LARS (*p* = 0.37). In univariate analysis, only postoperative complications occurred to be a risk factor for LARS development (*p* = 0.02, OR: 6.75, 95% CI 1.26–36.03). As univariate analysis identified only one risk factor of LARS we did not perform multivariate analysis of logistic regression.

Median Wexner score after the end of treatment was 7 in LaTME (IQR: 3–11) and 8 in TaTME group (IQR: 4–12) and did not differ significantly (*p* = 0.83). No risk factors were identified in univariate analysis (Table 2).

**Table 2:** Univariate risk factors analysis for LARS score and Jorge-Wexner scale

Univariate risk factors analysis for LARS scale			
	OR	95% CI	*p* value
Females vs. Males	0.84	0.26–2.68	0.767
Age	0.95	0.89–1.02	0.192
BMI	0.92	0.80–1.06	0.253
TaTME vs. LaTME	0.84	0.26–2.67	0.768
Depth	0,81	0.81–0.53	0.342
Radiotherapy	1.27	0.33–4.95	0.730
Anastomosis type (Stapled vs handsewn)	4,63	0.46–45.97	0.187
IAE	0.76	0.18–3.28	0.711
Operative time	1.00	0.99–1.01	0.712
Blood loss	0.998	0.995–1.002	0.406
Complications	6.75	1.27–36.03	0.025
cT	0.87	0.42–1.80	0.714
pT	1.21	0.74–2.00	0.451
AJCC	1.12	0.66–1.89	0.687
Adjuvant treatment	1.00	0.43–2.30	0.99
Univariate risk factors analysis for Jorge-Wexner scale			
	Parameter ± SD	*p* value	
Females vs. Males	0.562 ± 1.074	0.605	
BMI	0.120 ± 0.224	0.595	
TaTME vs. LaTME	0.723 ± 1.167	0.541	
Depth	0.511 ± 0.695	0.468	
Radiotherapy	1.129 ± 1.120	0.322	
Anastomosis (Stapled vs handsewn)	0.240 ± 1.036	0.817	
IAE	1.181 ± 1.390	0.402	
Operative time	0.019 ± 0.020	0.350	
Blood loss	0.005 ± 0.007	0.496	
Complications	1.268 ± 1.109	0.262	
cT	0.30 ± 0.970	0.755	
pT	1.080 ± 0.945	0.262	
AJCC	0.19 ± 0.710	0.785	
Adjuvant treatment	0.242 ± 1.520	0.757	

Notes: Abbreviations: *OR*, Odds ratio; *CI*, Confidence interval; *IAE*, Intraoperative adverse events

Differences in operative time: 212 (IQR: 180–250) minutes - LaTME vs. 252 (IQR: 190–300) - TaTME were not statistically significant (*p* = 0.14). Both groups were also comparable (*p* = 0.58) regarding blood loss: 100 (IQR: 50–100) ml and 100 (IQR: 50–250) ml in LaTME and TaTME groups, respectively. 3 (13%) intraoperative adverse events (IAE) occurred in LaTME group (1 intraoperative bleeding, 2 intraoperative anastomotic leakage which was repaired during the procedure). In TaTME group there were 4 (17%) IAE (2 purse string failures, 1 intraoperative anastomotic leak and 1 intraoperative bleeding). There were no statistical differences between the groups (*p* = 0.68).

Six patients from LaTME group and 5 patients from TaTME group suffered from postoperative complications, including four severe cases (Clavien-Dindo III-V) in each group. The summary of perioperative morbidity is presented in Table 3.

**Table 3:** Perioperative morbidity

Clavien-Dindo	LaTME	N (%)	TaTME	N (%)
V	–	0	–	0
IV	–	0	Intraabdominal abscess, sepsis	1 (4.5%)
IIIb	Anastomotic leakage (operative treatment)	2 (9%)	Anastomotic leakage	2 (9%)
	Postoperative ileus (operative treatment)	2 (9%)		
IIIa	–	0	–	0
II	High output stoma Anastomotic lekeage (conservative treatment)	1 (4.5%) 1 (4.5%)	Postoperative ileus (conservative treatment)	1 (4.5%)
I	–	0	–	0
Total		**6 (26%)**		**4 (17%)**

In 20 cases (86%) in TaTME group the quality of mesorectal excision was assessed as complete. In the remaining 3 (14%) cases, mesorectal excision was nearly complete. In LaTME group, 19 (83%) patients had complete and 4 nearly complete excisions. One patient in TaTME group (4,5%) had positive circumferential resection margin (CRM) and one patient in LaTME group (4,5%) had positive distal resection margin (DRM). These results of pathological outcomes are summarized in Table 4.

**Table 4:** Pathological outcomes

	LaTME	TaTME	*p* value
pT1/2/3/4	5/8/10/0	6/8/9/0	0.72
pN1/2	5/2	4/3	0.66
Quality of mesorectal excision: Complete/Nearly complete/Incomplete	19/4/0	20/3/0	0.52
Positive CRM	0 (0%)	1 (4.5%)	0.31
Positive DRM	1 (4.5%)	0 (0%)	0.31

Abbreviations: *CRM*, Circumferential resection margin; *DRM*, Distal resection margin

## Discussion

In our study we presented functional outcomes of low rectal resections using LaTME and TaTME techniques. Although the clinicopathological outcomes are acceptable, faecal continence is still far from satisfactory. Still, both techniques were comparable in terms of functional outcomes.

Oncological outcomes are of utter importance in rectal cancer treatment, yet patient’s quality of life should also be a matter of further discussion [19, 20]. Available studies do not focus on functional outcomes of TaTME. COLOR III – a Randomized Control Trial comparing LaTME and TaTME has functional assessment in the study protocol, but the results are expected to be released after 2020 [21]. Fecal incontinence is the element of LARS with the highest impact on quality of life in terms of social and professional life [22]. Surprisingly, irreversible loss of sphincter during abdomino-perineal (APR) resection is comparable to sphincter saving surgery regarding quality of life [22]. So far, we can present only short-term results, and we may expect that proper rehabilitation may improve the function of anal sphincters [23]. However, as pointed by other authors this improvement can be achieved only during first year after surgery and is more likely to be related to higher acceptance of symptoms than real recovery of continence [19].

The literature about functional outcomes of TaTME is extremely limited. Only one recent study published by Veltcamp et al. focused on patients’ quality of life [24]. Although authors failed to document significance (probably due to low study sample), the incidence of major LARS reached 30% in LaTME group and almost 60% in TaTME group. On the contrary, Elmore and Chen obtained acceptable results, however in this case only Wexner scale with median 8 points was used and authors stated that all patients were fully continent [25, 26]. In both of these studies the study group was very small including 6 cases. Therefore, further studies are required to evaluate TaTME functional outcomes.

In our research, only postoperative complications occurred to be a risk factor for LARS development. Bregendahl also points on anastomotic leakage as a factor predisposing for LARS [27]. Other authors underline the significance of neoadjuvant and adjuvant radiotherapy as a common risk factor for faecal incontinence [28, 29]. Also, defunctioning ileostomy may contribute to LARS [29]. What is important, extending time for ileostomy reversal beyond 6 months will also increase the rate of fecal incontinence [30]. Next, the distance of the tumor from the anal verge is a well known risk factor of LARS development [19]. Type of anastomosis may also affect functional outcomes in favor of stapled anastomosis versus hand-sewn [22]. As mentioned, Veltcamp published the first study comparing TaTME and LaTME, however, majority of patients were operated due to tumors of middle rectum, between 5 and 10 cm from the anal verge [24]. Our study is the first one to assess only low rectal resections, less than 5 cm from the anal verge.

Clinical and pathological outcomes of TaTME appear to be comparable to LaTME. Many authors suggested that TaTME may enable achieving wider CRM, although available meta-analyses present equivocal data [31–33]. Our series is too small to find evidence supporting this important aspect and this may be expected from the results of the ongoing, large randomized control trials [21, 34].

Our study has some obvious limitations. Firstly, the number of cases is limited which does not allow us to draw any strong conclusions. We also used questionnaires (Wexner scale and LARS score), but did not perform functional outcome analysis with electromyography or sphincter manometry, which could be useful in sphincter insufficiency detection. Having said that, LARS score and Jorge-Wexner scale are based on patient’s own view, which is helpful to understand and predict their quality of life [5, 12]. Furthermore, LaTME group consisted of patients with tumors localized slightly higher from the anal verge than in TaTME group. Nevertheless, both groups assessed only low rectal tumors, less than 5 cm from the anal verge.

## Conclusions

TaTME is a promising technique and provides comparable results to LaTME in regards to functional outcomes in low rectal cancer resections. Although clinical and pathological outcomes are satisfactory, the prevalence of LARS remains high and further evaluation of this technique is required.

## Data Availability

The datasets used and/or analysed during the current study are available from the corresponding author on reasonable request.

## Abbreviations

CRM: Circumferential resection margin
DRM: Distal resection margin
IAE: Intraoperative adverse events
LARS: Low anterior resection syndrome
LaTME: Laparoscopic total mesorectal excision
TaTME: Transanal total mesorectal excision
